# Religiosity and Psychotic Experiences: A Large Community-Based Study From Qatar

**DOI:** 10.1192/bjo.2022.260

**Published:** 2022-06-20

**Authors:** Peter Woodruff, Salma Khaled, Sanne Brederoo, Majid Alabdulla, Iris Sommer

**Affiliations:** 1 University of Sheffield, Sheffield, United Kingdom; 2 Qatar University, Doha, Qatar; 3 University Medical Center Groningen, Groningen, Netherlands; 4 Hamad Medical Corporation, Doha, Qatar

## Abstract

**Aims:**

We wished to explore associations between intrinsic religiosity, extrinsic (non-organizational (ENORG) and organizational (EORG)) religiosity and hallucinations phenomenology in a non-clinical Muslim population.

**Methods:**

We selected full-time students at Qatar University using systematic random sampling and administered the Questionnaire of Psychotic Experiences online. We modelled the effects of sociodemographic variables, anxiety, depressive symptoms, and religiosity measures, delusions on hallucinations severity and distress/impact in the past week, using structural equation modelling.

**Results:**

Direct-effects models supported ENORG religiosity's protective role against experiencing distress or negative impact on daily function from hallucinations. Intrinsic religiosity had positive indirect-effects on hallucinations distress/impact through depression, anxiety, and through EORG but negative (suppression) indirect-effects on hallucinations distress/impact through ENORG. Younger and married from lower socio-economic class participants had comparatively more severe hallucinations and more distress from them.

**Conclusion:**

We present evidence of differential associations between the religiosity types, socioeconomic and cultural groups, and past week distress/impact of hallucinations.

Our data support the importance of alignment between religious education and mental health and well-being education.

